# Acupuncture Enhances Effective Connectivity between Cerebellum and Primary Sensorimotor Cortex in Patients with Stable Recovery Stroke

**DOI:** 10.1155/2014/603909

**Published:** 2014-03-09

**Authors:** Zijing Xie, Fangyuan Cui, Yihuai Zou, Lijun Bai

**Affiliations:** ^1^Department of Neurology, Dongzhimen Hospital of Beijing University of Chinese Medicine, Beijing 100700, China; ^2^Dongzhimen Hospital Eastern Affiliated to Beijing University of Chinese Medicine, Beijing 101100, China; ^3^The Key Laboratory of Biomedical Information Engineering, Ministry of Education, Department of Biomedical Engineering, School of Life Science and Technology, Xi'an Jiaotong University, Xi'an 710049, China

## Abstract

Recent neuroimaging studies have demonstrated that stimulation of acupuncture at motor-implicated acupoints modulates activities of brain areas relevant to the processing of motor functions. This study aims to investigate acupuncture-induced changes in effective connectivity among motor areas in hemiparetic stroke patients by using the multivariate Granger causal analysis. A total of 9 stable recovery stroke patients and 8 healthy controls were recruited and underwent three runs of fMRI scan: passive finger movements and resting state before and after manual acupuncture stimuli. Stroke patients showed significantly attenuated effective connectivity between cortical and subcortical areas during passive motor task, which indicates inefficient information transmissions between cortical and subcortical motor-related regions. Acupuncture at motor-implicated acupoints showed specific modulations of motor-related network in stroke patients relative to healthy control subjects. This specific modulation enhanced bidirectionally effective connectivity between the cerebellum and primary sensorimotor cortex in stroke patients, which may compensate for the attenuated effective connectivity between cortical and subcortical areas during passive motor task and, consequently, contribute to improvement of movement coordination and motor learning in subacute stroke patients. Our results suggested that further efficacy studies of acupuncture in motor recovery can focus on the improvement of movement coordination and motor learning during motor rehabilitation.

## 1. Introduction

Although acupuncture has been widely used in rehabilitation of hemiplegic stroke patients in many parts of the world, the potential neural mechanism underlying the beneficial effect of acupuncture remains largely unknown. Recent neuroimaging studies have demonstrated that stimulation of acupuncture at motor-implicated acupoints modulates activities of brain areas relevant to the processing of motor signals [1–4]. These findings have shed some lights on the functional substrates of the purported therapeutical effect of acupuncture in stroke rehabilitation. However, the interactions within motor-related networks as well as its influence contributing to motor recovery induced by acupuncture have remained elusive.

Models of functional connectivity and effective connectivity can be used to describe the interactions between brain areas within brain network [5]. Recent neuroimaging studies have demonstrated that rehabilitative therapies can induce changes in effective connectivity of motor-related areas in stroke patients [6, 7], and rTMS or pharmacological treatments can also ameliorate stroke-induced deficits by enhancing effective connectivity within the motor network [8–10]. Acupuncture, which is a potentially effective therapy in stroke rehabilitation, has been reported to modulate resting state functional connectivity in the default mode and sensorimotor brain networks [11, 12]. However, recent studies have shown that functional connectivity and effective connectivity between different regions are both important and essential in detailing working mechanisms of the brain's functional architecture. The resultant model is primarily concerned with the directions of neural interactions and how one neural system exerts influence over another. Such information can be used to explore the specific role of a cortical region in a distributed system [5]. Changes in the pattern of normal cortical connectivity within and across hemispheres in stroke patients with motor deficits in the subacute phase have been discovered [13]. Moreover, James et al. found that improvements in motor performance were associated with enhanced interhemispheric communication [6]. These findings provide compelling rationales to investigate the acupuncture-induced changes in effective connectivity among motor areas in hemiparetic stroke patients.

Previous neuroimaging studies mainly focused on the functional specificity of motor-related acupoint on healthy subjects. According to the theory of traditional Chinese medicine, acupuncture is believed to exert various therapeutic effects by restoring the homeostatic balance [14]. Thus, acupuncture may have more specific effects on patient with a pathological imbalance compared to healthy subjects. In the present study, we investigated acupuncture stimulation at acupoint GB 34 in stroke patients and used healthy subjects as control condition. It has been reported that acupuncture can produce sustained effects even after the acupuncture manipulation being terminated [15, 16]. In this study, a nonrepeated event-related (NRER) design [12, 17] was employed to investigate effective connectivity changes after acupuncture administration. Based on the principle of temporal predictability, the Granger causality analysis can be used to explore effective connectivity between ROIs without any* a priori* specification of a network model [18–20]. In the present study, a multivariate Granger causality model was employed to obtain causal relations among multiple brain areas. This approach was based on a multivariate vector autoregressive (MVAR) model and allowed us to detect the simultaneous directional influences between multiple ROIs without any* a priori* specification of a network model. This approach has been successfully applied in many previous brain network studies [18–20]. We hypothesized that stroke patients may exhibit different models in effective connectivity within motor network involving both passive finger movements task and resting state, and acupuncture can induce relatively specific effects on the modulation of interactions within the motor network compared with healthy controls. In order to further understand the acupuncture mechanism, we conducted an fMRI study to identify acupuncture-induced changes in the interactions between motor-related areas that potentially facilitate motor recovery after stroke.

## 2. Materials and Methods

### 2.1. Subjects

From March 2012 to February 2013, a total of 9 patients who had ischemic strokes in the anterior circulation (7 males and 2 females, mean age 57.8 ± 9.9 years, mean days from first-onset stroke 53.6 ± 41.6 days, ranged from 18 to 122 days) were recruited from Beijing Dongzhimen Hospital. Inclusion criteria were as follows: (1) >2 and <12 weeks from the onset of ischemic stroke; (2) unilateral right-sided striatocapsular lesions; (3) Mini-Mental State Examination (MMSE) score ≥ 21 [21]; (4) moderate to severe motor deficits of the contralesional upper extremity, Motricity Index (MI) < 80; (5) right-handed individuals according to the Edinburgh Handedness [22]; (6) age range of 35–75 y. Exclusion criteria were as follows: (1) any clinically significant or unstable medical disorder, (2) bihemispheric or brain stem infarcts; (3) severe aphasia precluding communication, (4) any neuropsychiatric comorbidity other than stroke, and (5) standard contraindications for MRI such as non-MRI compatible implanted metallic devices. All patients recruited were scored on the following function measures on the same day as MRI: (i) Motricity Index (MI) for affected upper and (ii) lower limbs; (iii) NIHSS; (iv) Brunnstrom; (v) Modified Ashworth Scale; (vi) Barthel Index; (vii) Modified Rankin Scale.

An additional 8 healthy subjects were recruited from Dongzhimen Hospital as age-matched and sexually matched control subjects (6 males and 2 females; mean age 51.6 ± 4.8 years, all right-handed individuals). There was no significant difference in age between the patients and the healthy subjects (*P* = 0.132). All control subjects had no history of drug abuse, alcohol abuse, stroke, or other neurological or psychiatric diseases. This study was approved by the local Institutional Review Board and conducted in accordance with the Declaration of Helsinki, and full written informed consent was obtained from all subjects. All subjects were acupuncture naïve and patient characteristics are listed in Table 1.

### 2.2. Experimental Design

**Figure 1:**
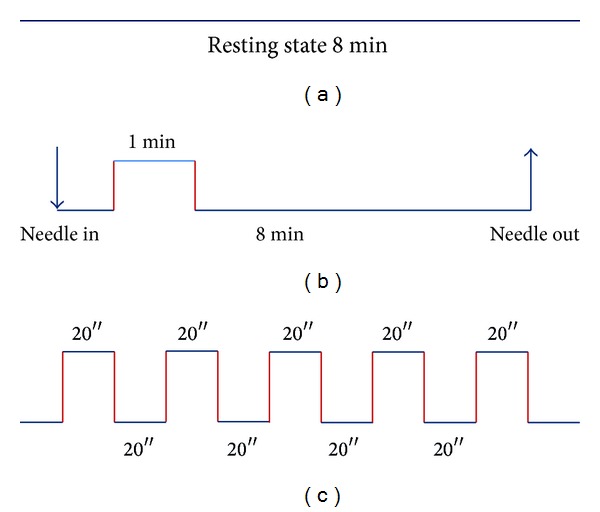
fMRI scan procedures. (a) Resting-state run lasted 8 min. (b) The acupuncture run employed the NRER-fMRI design paradigm, incorporating 1 min needle manipulation, preceded by 10 seconds rest and followed by 8 min rest scanning. (c) The motor task run employed a conventional block design in which five blocks of 20-second finger movement were alternated by five blocks of 20-second baseline, with 10 seconds rest in the beginning.

Patients and healthy subjects had the same MRI procedure. Eight patients underwent the experiment twice at an interval of two weeks. One patient only underwent the experiment once. Every control subject underwent the experiment once. Every experiment consisted of three functional runs including, successively, resting state scanning before and after acupuncture stimuli, and passive finger movement. Resting-state run lasted 8 min (Figure 1(a)). During the resting-state run, subjects were asked to lie motionless with their eyes closed, not to think of anything in particular, and not to fall asleep. Cushions were used to reduce head motions. The acupuncture run employed the NRER-fMRI design paradigm (Figure 1(b)), incorporating 1 min needle manipulation, preceded by 10 seconds rest and followed by 8 min rest scanning. The motor task run employed a conventional block design in which five blocks of 20-second finger movement were alternated by five blocks of 20-second baseline, with 10 seconds rest in the beginning (Figure 1(c)). The motor task consists of a repetitive movement in which the left thumb was passively opposed to the left index finger at the frequency rate of 1 Hz. After the three runs of scanning, all subjects were asked if they fell asleep during any of the runs.

Acupuncture was performed at an acupoint GB 34 on the left leg (Yanglinquan, located in a depression anterior and inferior to the head of the fibula). This acupoint is one of the most frequently used acupoints and proved to have various efficacy in the treatments of hemiplegic stroke in some previous researches [23, 24]. A sterile disposable 38-gauge sterling silver acupuncture needle (0.3 mm in diameter and 40 mm in length) was inserted vertically to a depth of 2-3 cm to deliver acupuncture stimulation. During acupuncture stimulation, the needle was rotated bidirectionally to an amplitude of approximately 180° for 1 min at a rate of 120 times per min by a balanced “tonifying and reducing” technique. All subjects were not informed of the presumed acupuncture effects. The procedure was performed by the same experienced and licensed acupuncturist on all subjects. Following the acupuncture run, subjects were presented with a 10-point visual analog scale (VAS) in which 0 = no sensation and 1–3 = mild, 4–6 = moderate, 7-8 = strong, 9 = severe, and 10 = unbearable sensation. Subjects were asked to rate the intensity of aching, pressure, soreness, heaviness, fullness, warmth, coolness, numbness, tingling, or dull or sharp pain they felt during the acupuncture run. Subjects were excluded from further analysis if they experienced sharp pain (greater than the mean by more than 2 standard deviations). None of the subjects experienced sharp pain among the 17 subjects.

### 2.3. Data Acquisition and Analysis

Imaging was performed on a 3.0 Tesla Siemens MRI Scanner in Radiology Department, Dongzhimen Hospital. A custom-built head holder and firm cushions were used to minimize the head motion. Functional scans were collected with sagittal sections parallel to the AC-PC plane. Thirty-two axial slices with coverage of the whole brain were obtained by using a T2*-weighted single-shot, gradient-recalled echo planar imaging (EPI) sequence. Acquisition parameters used in the functional scans were TE = 30 ms, TR = 2 s, flip angle = 90°; 3.5 mm slice thickness with 0.7 mm gap; 64 × 64 acquisition matrix with a field of view (FOV) of 225 mm × 225 mm. After acupuncture run, high-resolution structural images were acquired on each subject using a T1-weighted three-dimensional (3D) MRI sequence with a voxel size of 1 mm^3^ for anatomical localization. Acquisition parameters used in the structural scans were TR = 1.9 s, TE = 2.52 ms, matrix = 256 × 256, FOV = 250 mm × 250 mm, flip angle = 9°, slice thickness = 1 mm.

All images were preprocessed and analyzed using Statistical Parametric Mapping 5 (SPM5, http://www.fil.ion.ucl.ac.uk/spm). Images were first corrected for head movement using least square minimization. None of the subjects had excessive head movements (>1.5 mm) on any axis and head rotation more than one degree. Then the image data was further normalized to the MNI template and resampled at 2 mm × 2 mm × 2 mm. Finally, images were smoothed with a 6 mm full-width-at-half maximum (FWHM) Gaussian kernel to decrease spatial noise. Then these data were filtered by using a bandpass filter (0.01~0.08 Hz) to reduce the effect of low-frequency drift and high-frequency noise.

### 2.4. Definition of Regions of Interest

The motor task from each subject was entered into a general linear model (GLM) “fixed-effect” framework. BOLD signal change for motor task epochs compared to rest epochs was estimated at each voxel and individual t-maps were obtained. Then individual t-maps were entered into the “random effect” group analysis framework, and statistical maps were obtained (*P* < 0.05, FDR corrected) by one-sample *t*-test. Brain regions activated during motor task in different groups were determined by the statistical maps. As brain regions activated during finger movement in healthy controls have been studied in lots of previous researches [25–28], we selected brain regions activated during motor task in healthy group as regions of interest (*P* < 0.05, FDR corrected) for further effective connectivity. These motor-related brain regions included the bilateral declive, bilateral culmen, bilateral inferior frontal cortex, bilateral inferior parietal lobule, lateral nucleus of thalamus, bilateral superior temporal cortex, bilateral middle temporal cortex, left precentral cortex, left postcentral cortex, right precentral cortex, right postcentral cortex, precuneus, bilateral insula, bilateral posterior thalamus, bilateral anterior cingulate cortex, bilateral caudate nucleus, bilateral middle cingulate cortex, and substantia nigra. Taking into account the intersubject anatomical variance, ROIs were defined on individual anatomical map, and the obtained individual ROIs were registered to standard MNI space to get a group probabilistic anatomical map. Finally, ROIs were defined by using the standard Talairach-Daemon-based atlas. The time series within each ROI were selected, averaged across voxels, and normalized across subjects to obtain a single vector per ROI, separately for different conditions (resting state before and after acupuncture and motor task) in different groups. For bilaterally activated areas, time series were averaged.

### 2.5. Effective Connectivity Analysis Using mGCA

To detect the causal interactions among those selected ROIs during the three conditions, a multivariate autoregressive model (MVAR) of time series within each ROI was established. Directed transfer function (DTF) based on the principle of the Granger causality was computed in the multivariate autoregressive model [29]. To highlight the direct connections and reduce mediated influences, we calculated the partial coherence to evaluate the direct association between every two ROIs. An approach of surrogate data was employed to test the significance of the path weights. A null distribution of 2500 sets of surrogate data was generated and DTF was calculated from these datasets [30]. Finally, a one-tailed significance test was carried out to compare the DTF value from the original time series with null distribution (*P* < 0.01, corrected).

Comparison between the resting state and the postacupuncture resting state was performed on the path weight between any two ROIs. In this way, we obtained changes in causal influence within the motor-related network between rest state and postacupuncture rest state. To better understand, the dynamic characters of the network, the “in + out” degree of each node within the network, were calculated. “in + out” degree of a node was defined as the number of all edges connected directly with it, and notes with a standard deviation more than mean degree were considered as the hubs of the network. The hubs of the network were believed to exert important influence on the network dynamics.

## 3. Results

### 3.1. Psychophysical Results

In this study, all subjects reported de qi sensations in different intensity such as soreness, heaviness, and fullness during acupuncture stimulation. The sensation of fullness was reported most frequently among both patient and control groups. According to the subjects' reports, 85% of the patients and 50% of the healthy subjects experienced the sensation of fullness, while the second most prevalent sensation experienced was numbness in patient group and aching in healthy group. The sensation intensity (mean ± standard deviation) was 4.5 ± 1.9 in healthy group and 4.1 ± 1.9 in patients groups. Although the prevalence of various sensations was significantly different (*P* < 0.05) between the two groups, there was no significant difference in the intensity of the sensations experienced between two groups (*P* = 0.67).

### 3.2. mGCA Mapping Results

**Figure 2:**
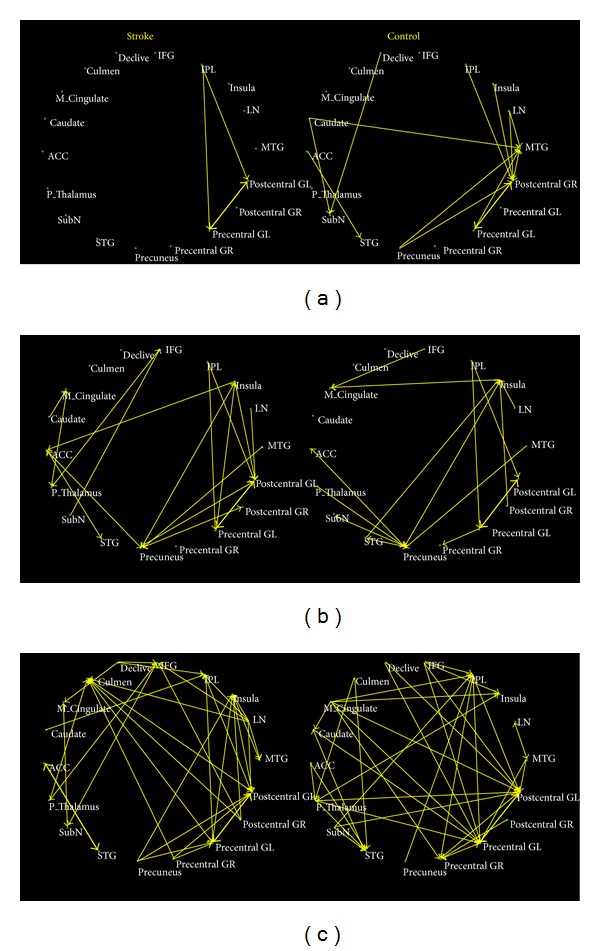
The visual description of causal connectivity between every two ROIs within the motor-related network with nodes representing the brain regions, edges thickness indicating the strength of influence, and arrows referring to directions of the influence. (a) Causal connectivity during motor task in stroke patients and controls. (b) Causal connectivity during resting state in stroke patients and controls. (c) Causal connectivity in postacupuncture state in stroke patients and controls.

A visual description of causal connectivity between every two ROIs within the motor-related network was carried out with nodes representing the brain regions, edges thickness indicating the strength of influence, and arrows referring to directions of the influence (Figure 2).

During motor task, the right postcentral gyrus and middle temporal gyrus served as the hubs of the network in healthy controls, receiving the most information inflows from cortical and subcortical brain regions, such as left precentral gyrus, precuneus, caudate nucleus, inferior parietal lobule, and lateral nucleus of thalamus. In stroke patients, the postcentral gyrus and precentral gyrus in left hemisphere turned into hubs of the network, receiving causal inflows from inferior parietal lobule and from each other (Figure 2(a)). This altered pattern of hubs showed a distinct shifting from right (contralateral) hemisphere to left (unaffected) hemisphere. In addition, the significant effective connectivities within the network in stroke patients were substantially reduced in comparison to those in healthy controls. Moreover, in stroke patients, there was a lack of significant connectivity in the subcortical regions such as the caudate nucleus and cerebellum.

Effective connectivity of the resting network was demonstrated, respectively, in different groups in Figure 2(b). Effective connectivity of the postacupuncture resting network was demonstrated, respectively, in different groups (Figure 2(c)). In healthy controls, left precentral gyrus, left postcentral gyrus, and inferior parietal lobule served as the hubs of the network, but in stroke patients, the hubs of the network included left precentral gyrus, left postcentral gyrus, insula, culmen, and the lateral nucleus of thalamus. It is interesting to note that although the acupuncture stimulation was performed on the left side, the left precentral and postcentral gyri, instead of the right precentral and postcentral gyri, served as hubs in the network, in both patients and healthy controls.

Changes in effective connectivity between resting and postacupuncture resting state were demonstrated, respectively, in different groups in Tables 2 and 3. In healthy controls, following acupuncture stimulation, the pre- and postcentral gyri received enhanced causal inflows from various brain regions including declive, substantia nigra, lateral nucleus of thalamus, and inferior parietal lobule. In stroke patients, following acupuncture stimulation, the pre- and postcentral gyri received enhanced causal inflows from culmen. Meanwhile, culmen also received enhanced inflows from right postcentral gyrus.

## 4. Discussion

In the current study, we investigated the effective connectivity changes involved in motor task in stroke patients to show motor-related connectivity deficits in stroke patients. Then, we assessed that acupuncture can induce relatively functional specificity modulation within the motor network in stroke patients, compared with the healthy controls.

### 4.1. Motor-Related Connectivity in Stroke Patients

In this study, we found not only remarkably reduced effective connectivity within the motor-related network but also a lack of connectivity between cortical and subcortical brain regions in stroke patients during motor task. Since effective connectivity represents the causal influence one brain region exerts on another [31], the lack of effective connectivity indicates the inefficient information transmission between motor-related regions. This observations is consistent with previous studies that the efficiency of information integration between motor-related regions was significantly decreased in stroke patients [32, 33]. Interconnected with cerebral cortex by multiple circuits, the target subcortical areas, such as the basal ganglia and cerebellum, have been traditionally regarded as important subcortical motor structures in mediating muscle tone changes and ensuring movement precision [34–36]. Therefore, the disruption of effective connectivity between subcortical and cerebral cortex may underly motor deficit in stroke patients.

### 4.2. Acupuncture-Induced Changes in Brain Connectivity

In healthy controls, significantly enhanced effective connectivity from various subcortical brain regions to sensorimotor cortex in both hemispheres was shown in postacupuncture resting state. By contrast, only one subcortical area (the culmen) showed enhanced effective connectivity with sensorimotor cortices in stroke patients. Moreover, the information transfers between the sensorimotor cortex and culmen were bidirectional. The target brain areas were more concentrated in stroke patients. Previous studies have reported that acupuncture can modulate the activity of sensorimotor areas [1, 2] and cerebellar structures [37, 38] at motor-implicated acupoint. However, few studies have demonstrated concentrated and bidirectional enhancements in causal inflows between the cerebellum and primary sensorimotor cortex in stroke patients. Interestingly, such increases in connectivity between the subcortical areas and sensorimotor cortex may compensate for the lack of connectivity between cortical and subcortical cortex in stroke patients when executing motor task.

Culmen is located in the anterior vermis, which is considered as part of spinocerebellum that receives sensory information from both the primary sensorimotor cortex and the periphery and sends modulation information back to the sensorimotor cortex and brain stem via deep cerebellar nuclei [39, 40]. In this way, cerebellum modulates the descending motor systems [39, 40]. Enhancement in effective connectivity between culmen and primary sensorimotor cortex could be related to more bidirectional information transfer in the cerebrocerebellar loops, which may lead to a stronger motor coordination effect of cerebellum on motor system. Cerebellum has also been considered as a key structure in feedback processing and storage of motor skill during motor learning, and motor learning mechanism is involved in both spontaneous recovery and rehabilitative trainings including constraint-induced movement therapy (CIMT) and impairment-oriented training (IOT) [41]. In fact, involvement of cerebellum in the process of motor recovery after stroke has been demonstrated in previous researches [42–45]. Increased functional connectivity between ipsilesional primary motor region and cerebellum persisted during the 6 months from onset in stroke patients [43]. Johansen-Berg et al. found that increases in activity of specific regions in the cerebellum and sensorimotor cortex correlated with improvement in motor function after motor rehabilitation and suggested that recovery after motor rehabilitation may be facilitated by changes of activity in cerebellum and sensorimotor cortices [46]. Moreover, an experimental study on rats has demonstrated that enhancement in the output of the dentatothalamocortical pathway improved motor recovery after strokes [47]. Taken together, the bidirectional increases in effective connectivity between cerebellum and sensorimotor cortex following acupuncture may contribute to the motor recovery after stroke by improving coordination of movement and motor learning. Accordingly, future efficacy studies of acupuncture in motor recovery can focus on the improvement of movement coordination and motor learning during rehabilitative trainings.

Previous connectivity study on tactile stimulation has demonstrated stronger functional connectivity of the primary and secondary somatosensory areas in contralateral hemisphere than in ipsilateral hemisphere following tactile stimulation [48]. Unlike tactile stimulation, there was a distinct shifting of the hubs from right hemisphere to left (ipsilateral) hemisphere in both stroke patients and healthy controls following acupuncture on the left side of the body, suggesting stronger influence of the ipsilateral sensorimotor cortex on the network dynamic. This finding coincides with the results of previous laser acupuncture studies suggesting that acupuncture effect is not only based on processing of afferent sensory information [49–51]. Furthermore, the ipsilateral central neural effect of acupuncture provides rationales for the classical needling method “opposing needling” according to which acupuncture can be performed on the opposite of the affected limbs.

Decreases in effective connectivity from ACC to precuneus during postacupuncture resting state in patients were also found in both healthy controls and stroke patients. This finding is in accordance with the results of previous researches which showed interrupted correlation between the precuneus and anterior cingulate cortex (ACC) during the poststimulation sate [52]. ACC is one of the important key nodes of the salience network, while precuneus plays a pivotal role in default mode network [53, 54]. Salience network is considered to identify the most relevant among internal and extra stimuli to guide behavior [53]. Therefore, the decrease of effective connectivity between the two networks may be due to acupuncture stimulation administration in both healthy controls and stroke patients.

According to the theory of Traditional Chinese Medicine, de qi is believed to be essential to the therapeutic effectiveness of acupuncture. One of the major limitations of the study was that it is hard to record for how long de qi sensation lasted after acupuncture stimulation. Another limitation was that the sample size in this study is not very large. In the future study, the use of time measurement in de qi sensation and a larger sample size might provide a clearer picture of the therapeutic effectiveness of acupuncture.

## 5. Conclusions

Acupuncture induced a concentrated and bidirectional enhancement in effective connectivity between cerebellum and primary sensorimotor cortex in stroke patients, which may contribute to improving coordination of movement and motor learning. Our results suggest that future efficacy studies of acupuncture in motor recovery can focus on the improvement of movement coordination and motor learning by combining with other rehabilitative trainings.

## Figures and Tables

**Table 1:** Clinical and demographic data.

Patient number	1	2	3	4	5	6	7	8	9
Age (years)	56	64	57	68	57	37	58	71	52
Gender	F	M	M	M	F	M	M	M	M
Localization of infarct	BG	IC	IC	CR	IC	IC	IC	IC	BG
Motricity index	0	60	14	72	23	60	34	76	76
	11	64	14	72	23	60	34	76	—
Rankin scale	4	1	2	2	4	2	3	2	2
	4	1	2	1	4	2	3	1	—
Barthel index	35	95	60	90	60	85	65	90	85
	40	95	65	85	60	85	75	90	—
NIHSS	14	3	9	5	8	7	7	3	5
	8	1	9	2	8	7	7	2	—
MMSE	22	30	27	29	22	30	30	24	30
	23	30	30	28	24	30	30	27	—
Brunnstrom	I	IV	II	II	I	V	II	V	II
	I	IV	II	III	I	V	II	V	—
Ashworth	0	1	1	0	0	2	2	0	0
	0	1	0	1	0	2	2	0	—

Abbreviations: BG: basal ganglia; IC: internal capsule; CR: corona radiate; NIHSS: National Institute of Health Stroke Scale; MMSE: Mini-Mental State Examination.

**Table 2:** Changes in effective connectivity during postacupuncture resting state in controls^a^.

Projecting regions	Receiving regions	*P *
Increased connectivity		
Declive	Postcentral GR	<0.05
MCC	Postcentral GR	<0.05
P_Thalamus	Postcentral GR	<0.01
IPL	Precentral GL	<0.05
MCC	Precentral GL	<0.01
Postcentral GR	Precentral GR	<0.05
SubN	Postcentral GL	<0.05
Insula	P_Thalamus	<0.05

Decreased connectivity		
Precuneus	ACC	<0.01
ACC	Precuneus	<0.01
STG	Insula	<0.05
Insula	Precuneus	<0.01
P_Thalamus	Precuneus	<0.01

^a^only *P* < 0.05 was listed in the table.

**Table 3:** Changes in effective connectivity during postacupuncture resting state in patients^b^.

Projecting regions	Receiving regions	*P *
Increased connectivity		
Insula	MTG	<0.05
MTG	Insula	<0.05
Culmen	Postcentral GL	<0.01
Precentral GR	Postcentral GL	<0.01
Postcentral GR	Culmen	<0.01
Culmen	Precentral GR	<0.05

Decreased connectivity		
ACC	Precuneus	<0.01
Insula	ACC	<0.01

^b^only *P* < 0.05 was listed in the table.
